# Effect of knowledgebase transition of a clinical decision support system on medication order and alert patterns in an emergency department

**DOI:** 10.1038/s41598-023-40188-4

**Published:** 2023-12-01

**Authors:** Weon Jung, Jaeyong Yu, Hyunjung Park, Minjung Kathy Chae, Sang Seob Lee, Jong Soo Choi, Mira Kang, Dong Kyung Chang, Won Chul Cha

**Affiliations:** 1 Department of Digital Health, Samsung Advanced Institute of Health Sciences and Technology (SAIHST), Sungkyunkwan University, Seoul, 06351 Korea; 2 Digital Innovation Center, Samsung Medical Center, Seoul, 06351 Korea; 3 Center for Health Promotion, Samsung Medical Center, Sungkyunkwan University School of Medicine, Seoul, 06351 Korea; 4 Department of Gastroenterology, Samsung Medical Center, Sungkyunkwan University School of Medicine, Seoul, 06351 Korea; 5 Department of Emergency Medicine, Samsung Medical Center, Sungkyunkwan University School of Medicine, 81, Irwon-Ro, Gangnam-Gu, Seoul, 06351 Korea

**Keywords:** Medical research, Virtual drug screening

## Abstract

A knowledgebase (KB) transition of a clinical decision support (CDS) system occurred at the study site. The transition was made from one commercial database to another, provided by a different vendor. The change was applied to all medications in the institute. The aim of this study was to analyze the effect of KB transition on medication-related orders and alert patterns in an emergency department (ED). Data of patients, medication-related orders and alerts, and physicians in the ED from January 2018 to December 2020 were analyzed in this study. A set of definitions was set to define orders, alerts, and alert overrides. Changes in order and alert patterns before and after the conversion, which took place in May 2019, were assessed. Overall, 101,450 patients visited the ED, and 1325 physicians made 829,474 prescription orders to patients during visit and at discharge. Alert rates (alert count divided by order count) for periods A and B were 12.6% and 14.1%, and override rates (alert override count divided by alert count) were 60.8% and 67.4%, respectively. Of the 296 drugs that were used more than 100 times during each period, 64.5% of the drugs had an increase in alert rate after the transition. Changes in alert rates were tested using chi-squared test and Fisher’s exact test. We found that the CDS system knowledgebase transition was associated with a significant change in alert patterns at the medication level in the ED. Careful consideration is advised when such a transition is performed.

## Introduction

Clinical decision support (CDS) system with electronic medical records systems (EMRs) has been widely used in clinical practice. If the system is appropriately established, it can improve the safety and effectiveness of care associated with medications^1–4^. On the other hand, if the alerting system is not managed systematically, it can trigger inappropriate alerts, high override rates, and alert fatigue^4,5^. The success of a CDS system is influenced by design factors such as user interface, automation and tiering, and users’ perception of its reliability^6–9^.

A knowledgebase (KB) plays a key role in ensuring the robustness of a clinical decision support system^8^. Medication-related knowledgebases are supplied by commercial vendors to assist end-users in prescribing medications. The knowledgebase vendor receives licensing fee as they can supply to multiple clients. With the help of a CDS system features and reasoning engine, the KB determines whether to fire an alert^10^. Due to the resource intensiveness of maintaining an up-to-date KB, many organizations use internally developed rules and/or rules provided by commercially available KBs, which show vast differences in their coverage and severity tiering^11,12^.

Exchanging KBs may be necessary for multiple reasons. Based on reasons for EMR transition, a KB transition could be due to the desire to improve the usability of the CDS system used quality of care, or financial status^13,14^. Many organizations yet have a relatively immature medication related CDS system, and KB plays an essential role in providing relevant drug alerts^8^. Similar to EMR transition, KB transition could result in work process interruption and poor compliance from users because adoption of a new KB requires a substantial change in users’ knowledge and behavior^15–17^.

To author’s knowledge, the transition of a KB as a whole has never been reported. The transition was implemented to all medications in the institute. Medication-related order and alert patterns in an emergency department before and after the transition were observed.

### Objective

The aim of this study was to analyze the effect of knowledgebase transition on medication-related orders and alert patterns in an emergency department.

## Materials and methods

This was a retrospective study that used data from an EMR. This study was approved by the Institutional Review Board (IRB) of Samsung Medical Center (IRB no. 2021-01-169). The requirement for informed consent was waived by the Institutional Review Board of Samsung Medical Center because de-identified data was used for analysis, and the study is retrospective and observational. All methods were performed and reported in accordance with “Strengthening the Reporting of Observational Studies in Epidemiology” (STROBE) guidelines^18^, and in accordance with the relevant guidelines and regulations. It was not appropriate or possible to involve patients or the public in the design, or conduct, or reporting, or dissemination plans of our research public were not involved in the design, or conduct, or reporting, or dissemination plans of this research.

### Study setting

The study was conducted in the emergency department (ED) of a tertiary urban academic medical center in Seoul, Korea. It is an acute care teaching hospital that receives approximately two million outpatients annually and has 1975 beds. Approximately 1000 doctors and 6000 nurses work in the institute. The ED has 69 beds with about 35 doctors, and the average annual patient volume ranges from 75,000 to 80,000. In Korea, only medical doctors can legally prescribe medication orders with very restricted exceptions.

### Electronic medical record system

The EMR used in this study was an internally developed system, which was rolled out in July 2016, replacing the previous internally developed EMR. The new EMR is a part of a hospital information system called Data Analytics and Research Window for Integrated kNowledge (DARWIN). DARWIN is a comprehensive system that contains computerized order entry from physicians as well as nurses, pharmacists, and billing and research support departments and even includes patient portal and web services.

### Computerized physician order entry and passive alert with inline text

**Figure 1:**
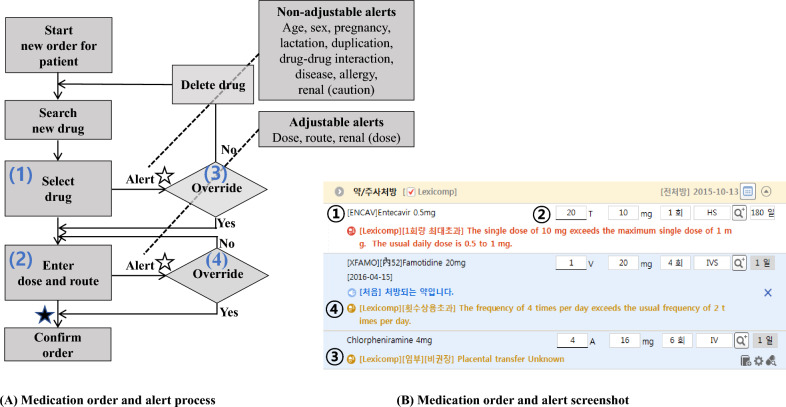
The order and alert process (**A**) and screenshot (**B**). Circled numbers in the process chart are matched with those in the screenshot. Hollow stars represent the point when the alert log is recorded. The alert log ID is replaced with the order log ID (solid star) when the order is overridden and confirmed.

Within DARWIN’s computerized physician order entry (CPOE) process, the prescription process is carried out as a sequence of actions. Once the process is initiated, a patient is selected, the diagnosis is confirmed, and orders for tests and medications are followed. When ordering a specific medicine, the medicine is searched for, and specifics such as doses, routes, and duration are entered. A schematic of this process is presented in Fig. 1. Alerts are categorized into two groups. The first group comprises non-adjustable alerts where users must change the drug in response to the alert because factors such as age and allergy cannot be changed. The other refers to adjustable alerts where users can change the specifics, such as dosage and route, to make the order consistent with the alert algorithm.

### Clinical decision support system design: passive inline text

A passive clinical decision support (CDS) system is integrated into the DARWIN’s computerized physician order entry (CPOE) for prescriptions. A passive CDS system is not likely to interfere in the work process of physicians. Although this type of alert may reduce alert fatigue, it may also result in decreased effectiveness of the CDS system^19,20^. As shown in Fig. 1, the alert appears before confirming the order.

### Clinical decision support system knowledgebase transition

In addition to the user interface, a knowledgebase (KB) for the CDS system was purchased commercially. Initially, the KB was supplied by Medi-Span (Wolters Kluwer Health, Philadelphia, PA, USA), with monthly updates. The types of alerts (domain) were related to age, allergy, disease, duplication, sex, lactation, pregnancy, dose, drug-drug interaction (DDI), and route. The KB was then changed to KIMS POC (KIMS, Seoul, Korea). The new KB covers a smaller range of medications and does not provide disease-drug and duplication alerts. The update interval was a week. In addition, the new KB was lower in cost than the previous KB (Table 1).

**Table 1:** Comparison of knowledgebases.

Knowledgebase	A medi-span	B KIMS POC
Interruption	Passive, Inline text	Passive, Inline text
Timing of alert	Before confirmation	Before confirmation
Database update period	Monthly	Weekly
Alert type (No. of covered drugs)		
Pregnancy, lactation, age, sex	1820	732
Disease-drug	5208	0
Allergy	261	2341
Dose	98,888	16,388
Route	1271	16,181
Drug-drug	3605	6282
Duplication	467	0
Renal	1820	732
Total	113,340	42,656

The knowledgebase transition was made from A to B. Coverage is described for each knowledgebase.

### Study population

Patients who visited the emergency department from January 2018 to December 2020 and were prescribed medication during their visit were eligible for inclusion in this study. Patients’ basic characteristics and clinical information regarding their visits to the emergency department were collected. The wash-out period was set from May 2019 to July 2019 (three months) to reflect the adjustment period for technical changes. All medication-related orders and alerts for these patients, and the basic information of the physicians were included in the analysis.

### Data extraction and preparation

Data on patients, physicians, and medication-related orders and alerts were extracted from the clinical data warehouse (CDW) of the study site. Patient data included age, sex, triage score, and visit date. The specifics of the physicians were also collected, including specialty department and position (trainee versus faculty [board-certified physicians]). Order data included patient identifier (ID), prescribing physician’s information, and prescribed medication information (order log ID, order date, drug name, dose, duration, and route). Alert data included patient ID, physician’s information, prescribed medication information, alert log ID, type of alert, and alert messages.

### Definition of orders

In this study, medication-related orders for all ED-based orders were included with a few exclusion criteria. Pro re nata orders were excluded because the final confirmation was made by nurses who did not receive medication-related alerts. Administrative order record data and fluid-type medications were excluded. The excluded orders did not generate any alerts.

The data comprised confirmed orders and intended (but withdrawn) orders. Multiple sequential alerts for a patient on the same medication provided by the same doctor were counted as a single order to reflect the intention of the physician. Order data captured dosage alteration within the same medication but failed to reflect drug changes after an alert has been generated. Given that a physician’s intention to change medication was unclear on hindsight, the new order was counted independently (Fig. 1).

### Definition of alerts and alert overrides

**Figure 2:**
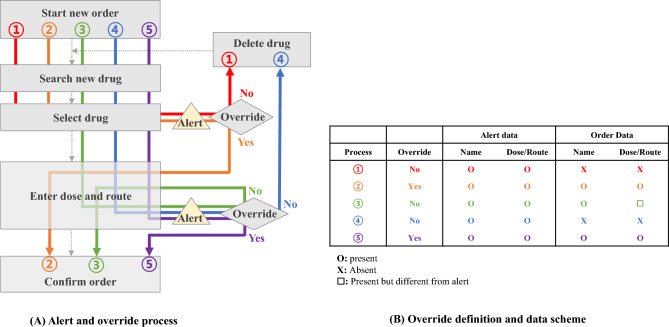
Alert override definition. Four representative alert-user interface cases are illustrated. Alert types are grouped into two (adjustable, non-adjustable). (**A**) Process of alert interaction. (**B**) Concept of data generation. Processes (1) and (4) reflect intended (but withdrawn) orders.

A set of definitions was required because an alert is generated before the order is confirmed. For non-adjustable alerts—age, sex, duplication, or DDI alerts—users have no other way to resolve the alert than to replace the selected drug. In this case, only the alert data remained without an order (Fig. 2). For adjustable alerts—dose or route alerts—users can either replace the drug to another or change the dose/route specifics to resolve the alert. Renal alerts were classified as non-adjustable or adjustable based on the alert messages.

A single event in the order data was a single confirmed medication-related order. Alert data were utilized to add intended (but withdrawn) order cases. To clarify a physician’s intention regarding the alert override, multiple alerts generated for a given drug were grouped appropriately based on a set of rules. If multiple adjustable alerts were given during the adjustment, only the final attempt was recorded. If a single drug led to multiple types of alerts, only one of each alert type was recorded.

A general rule was applied to define whether the alert was overridden. If a physician decided to delete the drug from the order after a non-adjustable or adjustable alert was given, processes (1) and (4) in Fig. 2, the physician was considered to not override the alert. If the physician decided to continue with the initial order after a non-adjustable or adjustable alert was given, processes (2) and (5), the physician was considered to have overridden the alert. Process (3) describes the case where a physician made adjustments to the drug dose or route after an adjustable alert was fired. If a physician followed the alert accordingly, the alert was not considered overridden.

### Data analysis and visualization

The study period was divided into two periods, A and B, based on the timing of the KB transition. The basic characteristics of the patients and alerts were described using simple statistics. Patient characteristics were compared for the two periods, and p-values were computed using a chi-squared test at a 0.001 significance level.

Changes in the order and alert patterns of commonly used drugs before and after the transition were examined. The alert rate (alert count divided by order count) and change in alert rate (period B alert rate minus period A alert rate) were computed. Drugs were then sub-grouped according to whether their alert rate increased or decreased after the transition compared to that before. The top 20 most commonly prescribed drugs during the study period were selected, and their alert patterns were examined. Changes in alert rate were tested using a chi-squared test and Fisher’s test at a 0.001 significance level. All analyses were performed using the statistical software, R (v4.0.3).

A direct comparison of alert types between the two vendors was not possible. Six out of 10 types of alerts—sex, pregnancy, lactation, disease, duplication, and route alerts—were not included by the period B vendor. Instead, the period B vendor provided a renal-type alert. Age-type alerts were initially not provided separately by the vendor used during period B. Instead, they were included as a subgroup under dose-type alerts. These subgroups were grouped prospectively as age-type alerts based on alert messages. The alert type composition of the alerts generated was broken down by month and visualized in conjunction with monthly override rates to observe the change in alert types over time.

## Results

**Figure 3:**
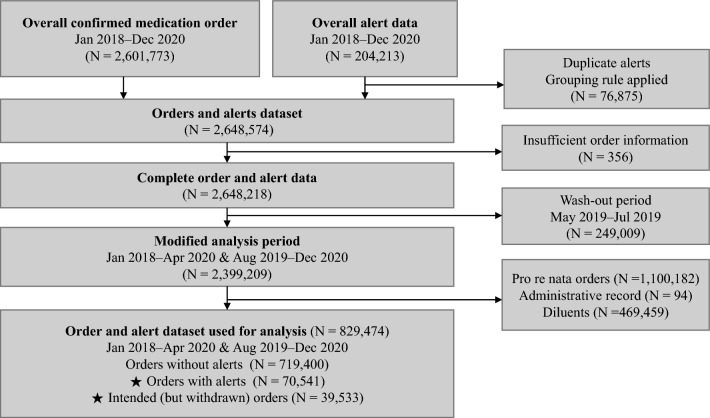
Medication order selection process. Medication-related order and alert data from January 2018 to April 2020 and August 2019 to December 2020 were used for statistical comparison. Both confirmed and intended (but withdrawn) orders were included in the analysis. The starred (★) alerts were later used to measure alert override rates.

The dataset used for analysis was built by integrating the confirmed medication-related order and alert data. Alert data includes intended (but withdrawn) order after an alert was fired. The detailed selection process is shown in Fig. 3. During the 33-month study period, 1325 physicians made 829,474 prescription orders; residents (60.9%) and fellows (24.3%) prescribed most of the orders. The rest of the orders were prescribed by assistant professors and professors. Medication orders include orders made during visit and at discharge. Alerts and orders used in our analysis were based on orders prescribed to patients in ED by physicians, not restricted to ED doctors, during our study period.

There were 168,849 patient visits to the ED during the study period. During period A, patients’ median age was 53 years (interquartile range [IQR]: 30–67 years), and during period B, it was 55 years (IQR: 32–68 years). Patient demographics were compared using chi-squared test at a 0.001 significance level. Despite the statistical difference in sex, age, Korean Triage and Acuity Scale score, and disposition between the two periods, the effect size was not large. Further patient descriptions are presented in Table 2.

**Table 2:** Patient demographics before and after the KB transition.

	Period A (Before transition) Jan 2018–Apr 2019	Period B (After transition) Aug 2019–Dec 2020	*p*-value*
	(N = 87,424)	(N = 81,425)	
Sex, N (%)			0.048
Female	43,662 (49.9)	40,273 (49.5)	
Male	43,762 (50.1)	41,152 (50.5)	
Age, N (%)			< 0.001
0–20 years	15,561 (17.8)	12,376 (15.2)	
20 to < 40 years	14,533 (16.6)	13,433 (16.5)	
40 to < 60 years	23,666 (27.1)	21,722 (26.7)	
≥ 60 years	33,664 (38.5)	33,894 (41.6)	
KTAS score^1^, N (%)			< 0.001
1 (most critical)	562 (0.6)	390 (0.5)	
2	4829 (5.5)	4706 (5.8)	
3	40,238 (46.0)	35,223 (43.3)	
4	36,922 (42.2)	37,208 (45.7)	
5 (least critical)	4873 (5.6)	3898 (4.8)	
Injury, N (%)			0.794
Non-injury	74,828 (85.6)	69,656 (85.5)	
Injury	12,596 (14.4)	11,769 (14.5)	
Disposition, N (%)			< 0.001
Discharge	60,470 (69.2)	54,518 (67.0)	
Admission	21,655 (24.8)	21,056 (25.9)	
Transfer	3085 (3.5)	2731 (3.4)	
LWBS^2^	1870 (2.1)	2758 (3.4)	
Death	344 (0.4)	362 (0.4)	

Data from May 2019 to July 2019 were removed as wash-out period.

^1^*KTAS* Korean triage and acuity scale.

^2^*LWBS* Leave without being seen.

*P-values were computed using a chi-squared test at a 0.001 significance level.

### Changes in alerts and alert overrides

Changes in alerts and related factors were observed before and after the KB transition. Alert rates (alert count divided by order count) for periods A and B were 12.6% and 14.1%, and override rates (alert override count divided by alert count) were 60.8% and 67.4%, respectively.

Order and alert patterns were observed at the medication level. The most commonly used medication based on the frequency of order in the ED during the study period was selected to evaluate the change in alert rates (period B alert rate minus period A alert rate). All drugs showed statistically significant differences in alert rates between the two periods, except for sodium bicarbonate 8.4% 20 mL (*p* = 0.013). Further information on order and alert patterns for the top 20 most commonly used medications is described in Table 3.

**Table 3:** Alert rates and alert rate change for the top 20 most common medication orders.

	Drug name, route* (Drug type)	Period A		Period B		Change in alert rate** %
		Order N	Alert N (%)	Order N	Alert N (%)	
1	Acetaminophen 650 mg, oral/IV (analgesic)	12,281	322 (2.6)	12,028	232 (1.9)	− 0.7
2	Ceftriaxone sodium 2 g, IV (antibiotic)	12,123	221 (1.8)	12,033	1551 (12.9)	11.1
3	Morphine HCl 10 mg, IV (opium alkaloid)	14,677	343 (2.3)	8795	137 (1.6)	− 0.7
4	Ketorolac 30 mg, oral/IV/IM (analgesic)	11,305	7037 (62.2)	6399	529 (8.3)	− 53.9
5	Famotidine 20 mg, oral/IV (H_2_ antagonist)	2923	41 (1.4)	14,715	739 (5)	3.6
6	Propacetamol 1 g, IV/IM (analgesic)	15,098	4032 (26.7)	2070	1291 (62.4)	35.7
7	Esomeprazole 40 mg, oral/IV (proton pump inhibitor)	9510	3794 (39.9)	7213	1926 (26.7)	− 13.2
8	Metoclopramide 10 mg, IV (antiemetic)	9087	843 (9.3)	7224	471 (6.5)	− 2.8
9	Tazoferan(R) 4.5 g, IV (antibiotic)	6632	126 (1.9)	7839	635 (8.1)	6.2
10	Metronidazole 500 mg, IV (antiprotozoal)	6567	33 (0.5)	6250	214 (3.4)	2.9
11	Ranitidine 150 mg, oral (H_2_ antagonist)	11,279	1952 (17.3)	1286	114 (8.9)	− 8.4
12	Chlorpheniramine 4 mg, IV/IM (antihistamine)	6396	1635 (25.6)	5698	14 (0.2)	− 25.4
13	Metoclopramide 5 mg, oral (antiemetic)	6247	58 (0.9)	4582	331 (7.2)	6.3
14	Sodium bicarbonate 8.4% 20 mL, IV (other)	3886	2 (0.1)	6482	3728 (57.5)	57.4
15	Ranitidine 50 mg, IV (H_2_ antagonist)	9105	220 (2.4)	1049	40 (3.8)	1.4
16	Levofloxacin 750 mg, oral/IV (antibiotic)	4016	556 (13.8)	4833	1521 (31.5)	17.7
17	Cetamadol(R) 325 mg/37.5 mg, oral (analgesic)	4641	108 (2.3)	4200	324 (7.7)	5.4
18	Scopolamine butylbromide 20 mg, IV/IM (anticholinergic)	4837	902 (18.6)	3870	3 (0.1)	− 18.5
19	Midazolam 5 mg, IV/IM (sedative)	4502	1172 (26)	3797	494 (13)	− 13
20	Hyoscine 10 mg, oral (anticholinergic)	4601	806 (17.5)	3667	10 (0.3)	− 17.2

Drug selection was based on the top 20 most commonly used drugs over the 33-month study period.

The alert rate was defined as the number of alerts fired over the order frequency count.

*Routes: oral, intravenous (IV), intramuscular (IM).

**Change in alert rate: Period B alert rate minus period A alert rate. P-values were computed using a chi-squared test and Fisher’s exact test.

A total of 644 drugs were used during both periods, and 296 drugs were prescribed more than 100 times during each period. Among these 296 drugs, 192 (64.5%) showed an increase in alert rate after the transition compared to the before transition value, and the median change was 11.10% (IQR, 4.90–25.00%). In contrast, for drugs that had a decrease in alert rate, the median change in alert rate was − 14.15% (IQR, − 24.75 to − 2.65%).

The top three most commonly prescribed drugs during the study period were acetaminophen (650 mg), ceftriaxone sodium (2 g), and morphine (10 mg). From the medications described in Table 3, sodium bicarbonate 8.4% 20 mL showed the greatest increase in alert rate (+ 57.4%), while ketorolac 30 mg showed the greatest decrease in alert rate (− 53.9%).

Between the two KBs, a vast difference in the alert content for the same drug was observed. For example, for ketorolac 30 mg, which showed the greatest decrease in alert rate, most alert types that were provided during period A were dose type (68.9%), while only 7.8% were dose type alerts during period B. The most frequent alert type during period B was DDI-type alerts (contraindicated) (83.6%). Age-type alerts (not recommended) were only present during period A, and DDI-type alerts were only present during period B. Ketorolac 30 mg override rates for periods A and B were 82.2% and 52.4%, respectively.

**Figure 4:**
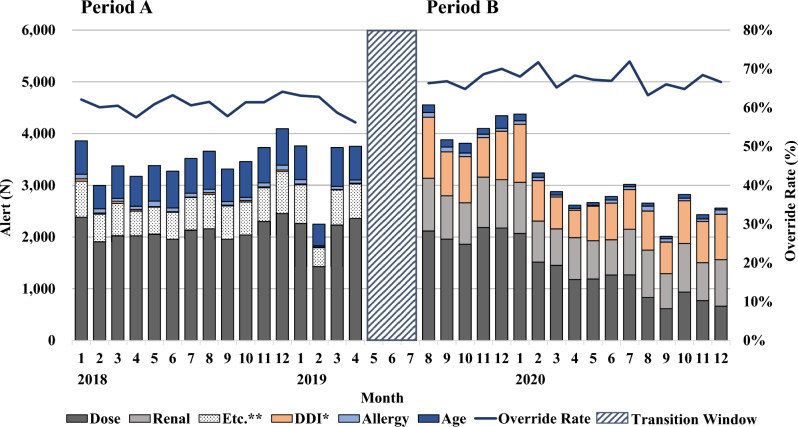
Monthly alert trend. * DDI: drug-drug interaction; **Etc. includes sex, pregnancy, lactation, disease, duplication, and route type alerts. The mentioned alert types were only present in period A; age-type alerts during period B were newly grouped based on alert messages indicating contraindications due to age. In February 2019, the medication clinical decision support system was turned off for two weeks for maintenance purposes. The decrease in dose-type alerts reflects dosage threshold alterations made in the alert firing rule during the study period.

A direct comparison between alert types was not possible, but we observed an increase in the share of adjustable alerts from period A (60.9%) to period B (70.0%). As shown in Fig. 4, there was a decrease in age-type alerts and an increase in DDI-type alerts. We can infer that there were fewer alert firing rules for age, while there were more alert firing rules for DDI in our new KB compared to that in the old KB. Meanwhile, alert firing rules for allergy-type seemed to be similar between the two KBs.

## Discussion

Medication-related alert patterns over a 33-month period before and after an abrupt knowledgebase (KB) transition between two commercial databases were described. To the best of our knowledge, there has been no report on the effect of KB transition. In this study, patients’ basic characteristics and alert patterns based on medication orders before and after the transition were described. Our study showed that higher alert and override rates were observed after the transition compared to that before the transition.

In our study, the knowledgebase used in phase B covered a smaller range of medications, did not provide ‘disease-drug’ and ‘duplication’ alerts, and was more economical. Yet, when the distinct number of medications that fired an alert and the distinct number of medications and alert types were compared between the two KB’s, both covered the medications commonly used in our ED. There were 883 distinct medications that fired an alert during phase A, while there were 881 distinct medications during phase B. Similarly, there were 1528 distinct medications and alert types during phase A, and 1492 during phase B. Although the KB used in phase A covered a wider range of medications and alert types, given the fact the range of medications used by the physicians did not change significantly, one could assume that the medications commonly used in our ED were mostly covered in both KB’s. Yet, our results showed an increase in both alert and override rates after the transition.

From Table 3, some of the medications show a substantial difference in alert rates before and after transition. Also, the order counts of ranitidine decrease greatly while famotidine increased greatly. This is because during our study, prescription of ranitidine was prohibited after detection of carcinogenic substance, and, thus, famotidine was prescribed instead. Sodium bicarbonate 8.4% 20 mL (57.4%), ketorolac 30 mg (53.9% decrease), propacetamol 1 g (35.7% increase), and chlorpheniramine 4 mg (25.4% decrease) showed alert rate differences greater than 20%.

Alert rate for sodium bicarbonate 8.4% 20 mL increased the most after transition. The knowledgebase used during phase A fired almost no alerts. However, KB used during phase B fired around 21,00 dose-type alerts and around 16,00 renal-type alerts. Dose-type alerts alerted that the medication should only be given 1–5 mg per order for adult patients. However, in the ED setting, sodium bicarbonate is often prescribed in higher amounts for severe acidosis patients or to patients before a CT scan. An alert was fired for above cases during phase B.

Alert rate for ketorolac 30 mg decreased the most after transition. Almost all alerts fired during phase A were dose-type and age-type alerts. Phase A KB fires an alert noting that ‘the single dose of 30 mg exceeds the maximum single dose of 15 mg’. This means that the KB used during phase A fired an alert every time ketorolac 30 mg was prescribed. Also, it fires an age-type alert that it is not recommended in geriatric patients. Whereas for phase B, most of the alerts were drug-drug alerts.

Alert rate increased the second most for propacetamol 1 g. Almost all alerts were pregnancy-type alerts during phase A, firing a message that ‘placental transfer is unknown’. On the other hand, almost all alerts were age-type alerts during phase B, which was fired when prescribed to pediatrics. Phase B knowledgebase follows the Korean guideline, and the Korean guideline does not recommend the use on children, because the safety of propacetamol on the use of pediatric patients have not yet been established. Yet, its use on pediatric patients is often used clinically.

It is difficult to predict the impact after a KB transition on paper, especially since the companies providing these knowledgebases do not open the alert-firing rules with the institute. However, if an institute does go through a transition, looking into the changes in alert and override rates before and after the transition may help in pinpointing to where to make amendments to the system. Fine-tuning alert-firing rules for the most commonly prescribed, alerted, or overridden medications based on clinical context may greatly impact the overall alert and override rates. Therefore, when considering a change in KB, it is extremely important to communicate with the actual uses, the physicians, and listen to their feedback after the transition is made. Revision to the default system based on department, patient status, institutional protocol, rules followed by the applicable country, and etc. may lower the alert and override rates.

With the integration of the medication CDS system, we aimed to effectively maintain or improve patient safety and physician practice. Although not observed in this study, it is important to look at the transition in terms of clinical effect, since it could result in work process interruption, as reported in previous studies on EMR transition^15–17^. Some measures to investigate the clinical effects include looking into adverse drug events, patient deaths attributed to drug errors, pharmacy interventions, or turnaround times for drug administration. Further studies on the clinical effects of this transition are needed.

Medication alert system should question the appropriateness of the alerts and overrides, especially when there are consistently high alert and/or override rates for certain medications. The aim is to fine-tune the system through step-by-step, systematic alterations to reduce inappropriate or excessive alerts, which may lessen alert fatigue experienced by physicians^4,8,12,21^. For a systematic development to be realizable, communication between physicians, pharmacists, and data processing team is recommended^22^. In addition, the development of a continuous monitoring system is recommended to sustain a better-quality medication CDS system^12^.

Increased alert and override rates after the transition may be a sign of excessive alert generation. One possible solution is to integrate machine learning or artificial intelligence (AI) technology into the system. This could enhance alert appropriateness and detect abnormal orders from orders that did not generate an alert, based on patient factor^4^. Further application of an AI-based system may provide recommendations for alternative medications after an alert has been provided, which may be a user-friendly tool for physicians^23^. Another additional benefit may be that an institute-trained dataset can incorporate any institute-, specialty-, or context-specific order protocols or patterns to the system^24,25^.

### Limitations

This study had some limitations. First, the study was performed in a single ED; therefore, these results may not be generalizable to other centers. Although widely used, the KBs used in our institute may not be used at other institutions, and alert logic from KBs may be tailored to adjust to any institution-specific needs. Second, the patients considered in the two periods may not be homogeneous. One of the suspected factors that may have contributed to this is the emergence of coronavirus disease 2019. Despite the statistical differences captured in some patient demographics, the effect size was not large. Lastly, this study did not examine the clinical outcomes following the transition and appropriateness of alerts and overrides. The appropriateness should be questioned for medications that generated high volumes of alerts and overrides. Further studies are needed to examine the clinical outcomes and appropriateness of a KB transition.

## Conclusions

Our study can contribute as a reference and provide knowledge to other institutions considering a transition or a major change in knowledgebase. In this study, we found that a knowledgebase transition was associated with a significant change in order and alert patterns in an emergency department. Careful consideration before execution is advised when such a transition is carried out.

## Data Availability

Data was available in study site clinical data warehouse. The datasets generated and analyzed during the current study are not publicly available due dataset includes although is de-identified, part of patient information, but are available from the corresponding author on reasonable request.
